# Novel Mutations in the *MC2R* Gene in a Patient With Familial Glucocorticoid Deficiency (FGD): A Case Report and Functional Study

**DOI:** 10.1002/mgg3.70242

**Published:** 2026-07-15

**Authors:** Ni Zhen, Yonghui Tao, Chuanyin Li, Wenli Lu

**Affiliations:** ^1^ Department of Pediatrics Ruijin Hospital Affiliated to Shanghai Jiao Tong University School of Medicine Shanghai China; ^2^ Department of Endocrine and Metabolism Ruijin Hospital Affiliated to Shanghai Jiao Tong University School of Medicine Shanghai China; ^3^ State Key Laboratory of Molecular Biology, Center for Excellence in Molecular Cell Science Shanghai Institute of Biochemistry and Cell Biology, Chinese Academy of Sciences Shanghai China; ^4^ Department of Colorectal Surgery and Oncology The Second Affiliated Hospital, Zhejiang University School of Medicine Hangzhou Zhejiang China

**Keywords:** adrenocortical dysfunction, cAMP, familial glucocorticoid deficiency, MC2R

## Abstract

**Purpose:**

Familial glucocorticoid deficiency (FGD) is a rare autosomal recessive disorder characterized by resistance to adrenocorticotropic hormone (ACTH), leading to isolated glucocorticoid deficiency. This study aims to identify the genetic basis of FGD in a Chinese patient and investigate the functional consequences of the detected *MC2R* mutations.

**Methods:**

Whole‐exome sequencing was performed to detect pathogenic variants in the *MC2R* gene. The effects of these mutations on *MC2R* mRNA and protein levels were analyzed using qPCR and immunoblotting. Additionally, a luciferase reporter assay was conducted to evaluate ACTH‐induced cyclic adenosine monophosphate (cAMP) signaling.

**Results:**

The patient was found to carry compound heterozygous mutations in *MC2R* (p.Leu151Pro and p.Glu28*), inherited from the father and mother, respectively. Functional studies revealed that these mutations led to reduced *MC2R* mRNA and protein expression. Furthermore, the luciferase assay demonstrated that these variants attenuated ACTH‐induced cAMP signaling.

**Conclusion:**

Novel pathogenic mutations in the *MC2R* gene were identified, and their functional impact was characterized. These findings provide insights into the molecular mechanisms underlying FGD and contribute to the expanding genetic spectrum of the disease.

## Graphical abstract

Compound heterozygous MC2R variants were identified and shown to reduce receptor expression and ACTH‐induced cAMP signaling, confirming their pathogenic role in familial glucocorticoid deficiency.

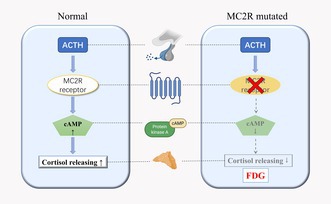

## 1 Introduction

Familial glucocorticoid deficiency (FGD) is a rare genetic disorder that typically manifests in childhood and, due to its nonspecific clinical presentation, diagnosis is often delayed. As a form of adrenocorticotropic hormone (ACTH) resistance syndrome, FGD is characterized by isolated glucocorticoid deficiency with preserved mineralocorticoid function. Common clinical manifestations include recurrent episodes of hypoglycemia and increased susceptibility to infections, often accompanied by hyperpigmentation. Recent studies have expanded the spectrum of genetic variants associated with FGD, including non‐classical mechanisms such as uniparental disomy and promoter region deletions (Müller‐Nedebock et al. 2025). To date, seven genes have been implicated in FGD, including *MC2R*, *MRAP*, *STAR*, *NNT*, *TXNRD2*, *MCM4*, and *SGPL1*.

FGD was first described in detail in 1959 by Shepard, with subsequent studies further characterizing the condition. However, the genetic basis of the disease remained unknown until 1993, when the first mutation in the MC2R (MIM #607397) coding sequence was identified; the molecular mechanisms underlying this disease remain incompletely understood. From that moment on, the number of reports involving sequencing of MC2R, which is located on the short arm of chromosome 18 (18p11.21‐pter) (Vamvakopoulos et al. 1993), increased considerably, leading to a better understanding of its structural and functional properties. The gene MC2R, also known as ACTHR, encodes a member of the five‐member G‐protein‐coupled melanocortin receptor family. *MC2R* is the smallest G protein‐coupled receptor (GPCR) comprising 297 amino acids and functioning as a seven‐transmembrane domain receptor. All this information provides clear links between genetic cause and physiological effect. Approximately 25% of patients with isolated FGD have mutations or deletions within MC2R, the cause of FGD1. Therefore, the disorder caused by a faulty MC2R gene was designated as FGD type I (OMIM #202200) (Heshmatzad et al. 2020).

In this study, two novel MC2R variants were found in one patient presenting with hyperpigmentation, hypoglycemic attacks, and convulsions in infancy and abnormally high stature in follow‐ups. Given the potential severity of FGD, which can result in life‐threatening complications such as coma, misdiagnosis remains a significant concern (Özbek et al. 2021). To further elucidate the pathogenicity of these variants, functional studies were conducted to assess their impact on ACTH signaling.

## 2 Materials and Methods

### 2.1 Editorial Policies and Ethical

A Chinese female neonate (2 days old) patient harboring novel MC2R gene mutations was enrolled in this study at Ruijin Hospital. The study was approved by the Institutional Review Board of Ruijin Hospital. Written informed consent was obtained from the parents.

### 2.2 Molecular Investigations

Genomic DNA was extracted from peripheral blood leukocytes using a DNA extraction kit (Tiangen, China). Whole‐exome sequencing (WES) was performed by a third‐party agency (Guangzhou KingMed Diagnostics Group Co. Ltd.) using the Illumina platform (USA), generating 150‐bp paired‐end reads. The average coverage depth of the target regions was ≥ 90×. Variant interpretation was conducted according to standard clinical protocols. Variants identified through WES were validated by Sanger sequencing. Variant pathogenicity was assessed according to the ACMG/AMP guidelines (Nykamp et al. 2017). Additional laboratory evaluations were conducted by the Department of Laboratory Medicine, Shanghai Ruijin Hospital.

### 2.3 Plasmid Construction

The plasmids pGL3‐miniCMV and pRL‐TK were generously provided by Professor Ronggui Hu (Chinese Academy of Sciences, Shanghai, China). Full‐length MC2R was amplified from genomic DNA of HEK293T cells and inserted into the pCDNA3.0‐Flag plasmid (provided by Professor Ronggui Hu), as the MC2R gene consists of a single exon. The pCDNA3.0‐MRAP‐HA was purchased from CellResearcher Biotechnology Co. Ltd. (Shanghai, China). Site‐directed mutagenesis was performed to introduce MC2R mutations as described previously (Xu et al. 2018). A synthetic cAMP response element consisting of six repetitive sequences (GENEWIZ, China) was inserted into the pGL3‐miniCMV vector to generate the pGL3‐CREB construct.

### 2.4 Cell Culture and Transfection

Human HEK293T, HeLa, and mouse Y1 cell lines were generously provided by Professor Ronggui Hu (Chinese Academy of Sciences, Shanghai, China). Cells were maintained in Dulbecco's Modified Eagle Medium (DMEM; Life Technologies, USA) supplemented with 10% fetal bovine serum (FBS), 100 U/mL penicillin, and 100 μg/mL streptomycin (all from Gibco, USA) under a humidified atmosphere of 5% CO_2_ at 37°C. Plasmid transfection into HEK293T cells was carried out using Lipofectamine 2000 (Life Technologies, USA) following the manufacturer's instructions.

### 2.5 RNA Extraction, Reverse Transcription PCR (RT‐PCR) and Quantitative PCR (qPCR)

Total RNA was extracted from cells using a total RNA extraction kit (Tiangen, China). Complementary DNA (cDNA) was synthesized with ReverTra Ace qPCR RT Master Mix (Toyobo, Japan). For RT‐PCR, MC2R and GAPDH were amplified using 2 × PCR mixture (Tiangen) and analyzed by agarose gel electrophoresis. The RT‐PCR reactions were performed using the following primers: MC2R, Forward: 5′‐CTGTCCTCGTGTGGTTTTGCCG‐3′, Reverse: 5′‐GATGACCGTAAGCACCACCACA‐3′; GAPDH, Forward: 5′‐GGGCGCCTGGTCACCAGGGC‐3′, Reverse: 5′‐GGACTCCACGACGTACTCAGC‐3′.

Quantitative PCR (qPCR) was conducted using SYBR Green Master Mix (Toyobo, Japan) on a CFX96 real‐time PCR system (Bio‐Rad, USA) following the manufacturer's protocol. The relative expression levels of target genes were normalized to GAPDH using the 2^−ΔΔCt^ method. The qPCR reactions were performed with the following primers: MC2R, Forward: 5′‐CACAGCCGATGACATCATCGACTC‐3′, Reverse: 5′‐CCGTAAGCACCACCACAGTGC‐3′; GAPDH, Forward: 5′‐CATGGAGAAGGCTGGGGCTC‐3′, Reverse: 5′‐GTGCAGGAGGCATTGCTGATG‐3′.

### 2.6 Immunoblotting Analysis

Immunoblotting analyses were performed as follows. Briefly, HEK293T cells, either transfected with plasmids or untransfected, were lysed in RIPA buffer (50 mM Tris–HCl, 150 mM NaCl, 5 mM EDTA, 0.1% sodium dodecyl sulfate [SDS], and 1% NP‐40, pH 7.6) supplemented with protease inhibitor cocktails (Selleck, China). The lysates were denatured at 100°C for 15 min in 1× SDS‐PAGE loading buffer and then subjected to SDS‐PAGE, followed by transfer onto a PVDF membrane (Bio‐Rad, USA). The membranes were incubated with primary antibodies against GAPDH (1:8000, 60004‐1‐Ig, Proteintech, China) and MC2R (1:500, ab135908, Abcam, USA). Subsequently, the membranes were incubated with HRP‐conjugated secondary antibodies, either goat anti‐mouse IgG (1:5000, SA00001‐1, Proteintech) or goat anti‐rabbit IgG (1:5000, SA00001‐2, Proteintech). Protein signals were detected using the Tanon 5200 Imaging System (Tanon, China). All transfections were performed in three independent replicates, with each replicate analyzed by immunoblotting.

### 2.7 Luciferase Reporter Assay

HEK293T cells were seeded into 24‐well plates at a density of 1 × 10^6^ cells/well. After overnight incubation, cells were transiently transfected with pGL3‐miniCMV or pGL3‐CREB along with other vectors (pRL‐TK and either wild‐type MC2R, MC2R‐p.Leu151Pro, or MC2R‐p.Glu28*). At 36 h post‐transfection, cells were treated with 20 nM adrenocorticotropic hormone (ACTH, Sigma, USA) for 30 min, lysed with 5× passive lysis buffer, and analyzed using the Dual‐Luciferase Reporter Assay System according to the manufacturer's instructions (Promega, USA).

### 2.8 Statistics

Data were analyzed using a two‐tailed unpaired *t*‐test or one‐way ANOVA followed by Tukey's post hoc test in GraphPad Prism 7. Statistical significance was set at *p < 0.05, while **p < 0.01 was considered highly significant.

## 3 Results

### 3.1 Identification of MC2R Variants

**FIGURE 1:**
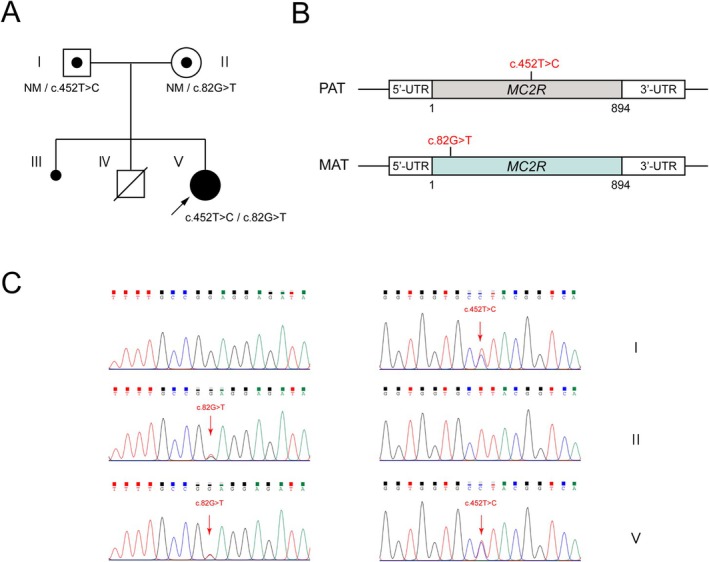
Novel MC2R variants in a patient with familial glucocorticoid deficiency (FGD). (A) Pedigree of a family carrying novel c.452T>C and c.82G>T variants in the human MC2R gene. Squares represent male family members, while circles represent females. Black‐filled symbols denote individuals diagnosed with FGD. Symbols with a black dot inside indicate asymptomatic carriers. Gray‐filled symbols represent individuals with suspected but unconfirmed FGD. A black dot represents an abortion with an unknown genotype, while a square with a diagonal bar indicates a deceased individual with an unknown genotype. NM denotes a wild‐type allele. The proband is marked by an arrow. (B) Schematic representation of MC2R gene variants, highlighting that the gene contains a single coding exon. (C) Partial sequencing chromatograms of the MC2R gene from the FGD patient and her family members.

The pedigree of the family carrying MC2R variants and the results of mutation analysis by whole‐exome sequencing (WES) and Sanger sequencing are presented in Figure 1. The proband inherited the c.82G>T (p.Glu28*) variant from her mother and the c.452T>C (p.Leu151Pro) variant from her father. According to the ACMG/AMP guidelines, these mutations were classified as pathogenic and likely pathogenic, respectively (Table 1).

**TABLE 1:** *MC2R* gene mutations in the proband.

Gene	Inheritance	Mutation	Genetic origin	Variant evidence	ACMG classification
MC2R	AR	c.82G>T; p.(Glu28*)	Mother	PVS1 + PM2 + PP3 + PP4	Pathogenic
		c.452T>C; p.(Leu151Pro)	Father	PM2 + PM3 + PP3 + PP4	Likely pathogenic

### 3.2 Clinical Characteristics of the Proband

A female infant of Han ethnicity was born at full term via vaginal delivery, with a birth weight of 3200 g and a length of 50 cm. She was the third child of non‐consanguineous parents. Her Apgar scores remained normal up to 15 min after birth. The family history was notable for the death of her elder brother at one year of age, who had presented with generalized hyperpigmentation since birth and died from infection‐related complications (Figure 1).

On the second day of life, the neonate developed convulsions, moaning, poor sucking reflex, hyperbilirubinemia, and severe hypoglycemia (1.5 mmol/L). Physical examination revealed hypertelorism, normal female genitalia with generalized hyperpigmentation, intact neonatal reflexes, and no signs of achalasia or alacrima.

Laboratory tests showed an elevated plasma ACTH level (> 2000 pg/mL, 08:00), a markedly reduced cortisol level (0.05 μg/dL, 08:00), and a DHEA‐S level of 2.0 μg/dL. Thyroid function and brain MRI findings were unremarkable. The patient received intravenous dextrose infusion, leading to clinical improvement and resumption of adequate oral intake. Given the clinical presentation and laboratory findings consistent with adrenal insufficiency, oral hydrocortisone therapy (15 mg/m^2^/day) was initiated.

During follow‐up, hyperpigmentation gradually improved. At eight months of treatment, ACTH level decreased to 388 pg/mL (08:00) and cortisol increased to 2.77 μg/dL (08:00). Electrolyte levels remained within the normal range (K 5.15 mmol/L, Na 136 mmol/L). The hydrocortisone dosage was adjusted according to body weight. At 2.5 years old, the patient remained clinically stable without recurrence of hypoglycemia or seizures. Her skin hyperpigmentation has improved. Hormone evaluation showed a further decrease in ACTH (286 pg/mL, 8:00) and an increase in cortisol levels (6.53 μg/dL, 8:00). At 6 years of age, the patient continued to show stable metabolic and hormonal control, with ACTH level of 220 pg/mL (8:00) and cortisol level of 6.7 μg/dL (8:00). Serum electrolyte levels remained in the normal range. Regarding growth, her height was 102 cm (+2.86 SD) at 2.5 years of age and 117.7 cm (+0.24 SD) at 6 years of age. Hyperopia was noted during follow‐up. (Table 2).

**TABLE 2:** Clinical and laboratory characteristics of the proband with MC2R mutation.

	At onset	After treatment		
Age	2 days	8 months	2.5 years	6 years
Weight (kg)	3.2	—	16 (+1.90 SD)	21 (+0.22 SD)
Height (cm)	50	—	102 (+2.86 SD)	117.7 (+0.24 SD)
Mode of presentation	Moderate hyperpigmentation; Poor sucking reflex; Wide‐set eyes; Low nasal bridge	Mild hyperpigmentation; Wide‐set eyes; Low nasal bridge	Mild hyperpigmentation; Wide‐set eyes; Low nasal bridge; Hyperopia	Minimal hyperpigmentation; Wide‐set eyes; Low nasal bridge; Hyperopia; Strabismus
Tanner stage	B I	B I	B I	B I
Pubarche stage	PH I	PH I	PH I	PH I
Glycemia (5–8 mmol/L)	1.5	6.3	5.1	5.3
ACTH (08:00 7–65 pg/mL)	> 2000	388	286	220
Cortisol (08:00 6.4–22.8 μg/dL)	0.05	2.77	6.53	6.7
Na (135–155 mmol/L)	138	136	137	135
K (3.5–5.5 mmol/L)	4.5	5.15	4.6	4.7
IGF‐1 (ng/mL)	—	—	157	192
Auxiliary examination	Patent ductus arteriosus	—	—	—
	Mitral regurgitation			
	Tricuspid regurgitation; Hydrocephalus			

No other family members, apart from the deceased sibling, have a history of unexplained death.

### 3.3 Variants of MC2R Attenuated Its Protein Levels

**FIGURE 2:**
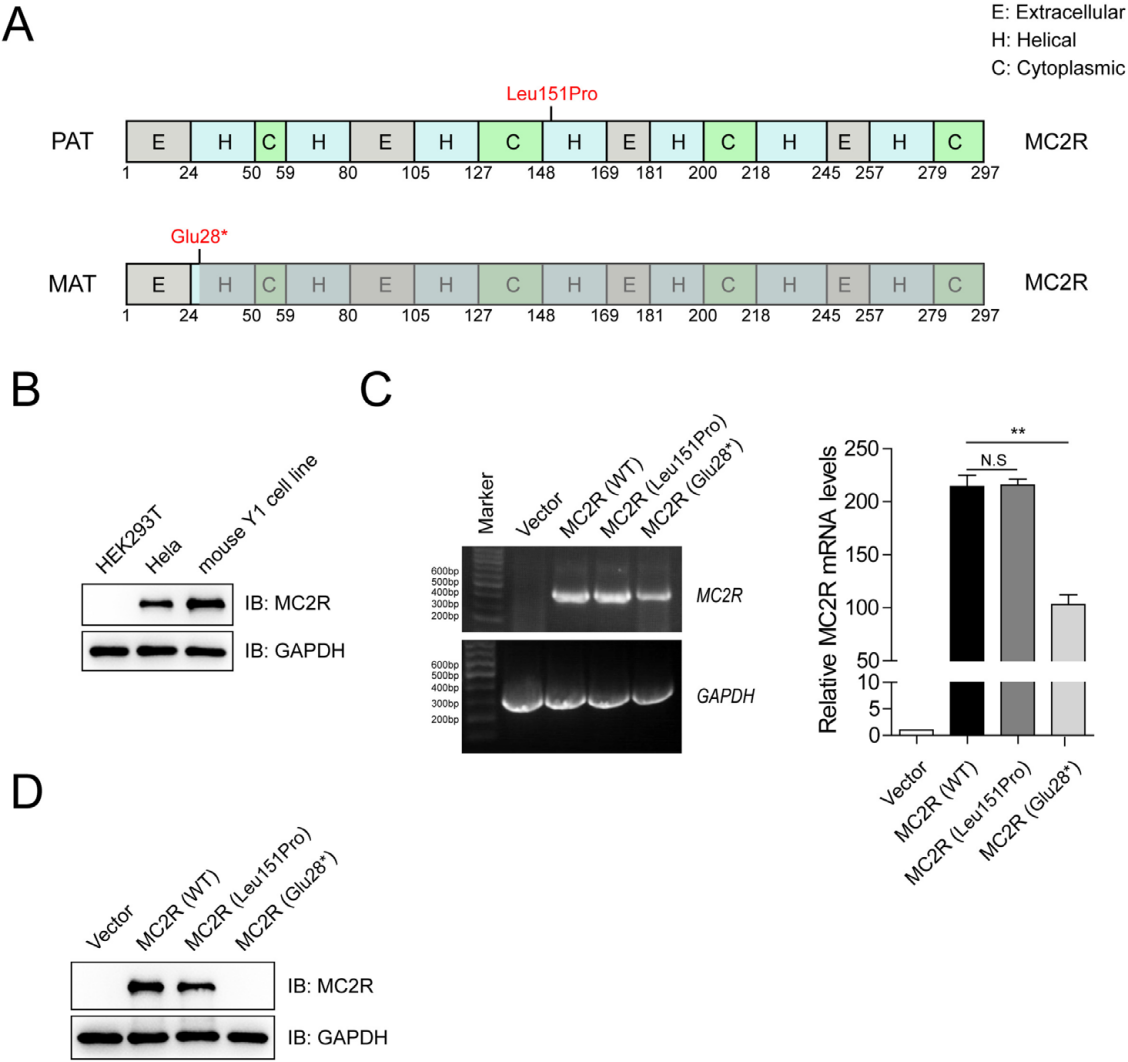
Variants of MC2R attenuated its protein levels. (A) Schematic representation of human MC2R protein variants analyzed in this study. E: Extracellular domain, H: Helical region, C: Cytoplasmic domain. (B) Immunoblotting analysis of MC2R protein expression in different cell lines. (C) mRNA expression levels of wild‐type and mutant MC2R analyzed by RT‐PCR (left) and qPCR (right). HEK293T cells were transfected with plasmids encoding Flag‐tagged empty vector, wild‐type MC2R, or the indicated mutant constructs. Total RNA was extracted, reverse‐transcribed into cDNA, and analyzed for MC2R expression. Data are presented as mean ± SD. *p* < 0.01, highly significant; n.s., not significant. (D) Immunoblotting analysis of MC2R protein expression in HEK293T cells transfected with an empty vector, Flag‐tagged wild‐type MC2R, or its mutants. All experiments were performed in three independent replicates.

Two MC2R variants, MC2R (p.Leu151Pro) and MC2R (p.Glu28*), were analyzed in this study (Figure 2A). As shown in Figure 2B, no endogenous MC2R protein signal was detected in HEK293T cells, making them suitable for further analysis. Vectors carrying either wild‐type MC2R or its mutant forms were transfected into HEK293T cells. Among them, only MC2R (p.Glu28*) exhibited significantly reduced mRNA levels. Immunoblotting analysis further demonstrated that the protein level of MC2R (p.Leu151Pro) was lower than that of the wild‐type receptor. In contrast, no detectable MC2R protein signal was observed in HEK293T cells transfected with MC2R (p.Glu28*), as only the first 27 amino acids before the premature stop codon could be translated (Figure 2C,D).

### 3.4 Variants of MC2R Attenuated ACTH‐Induced cAMP Response

**FIGURE 3:**
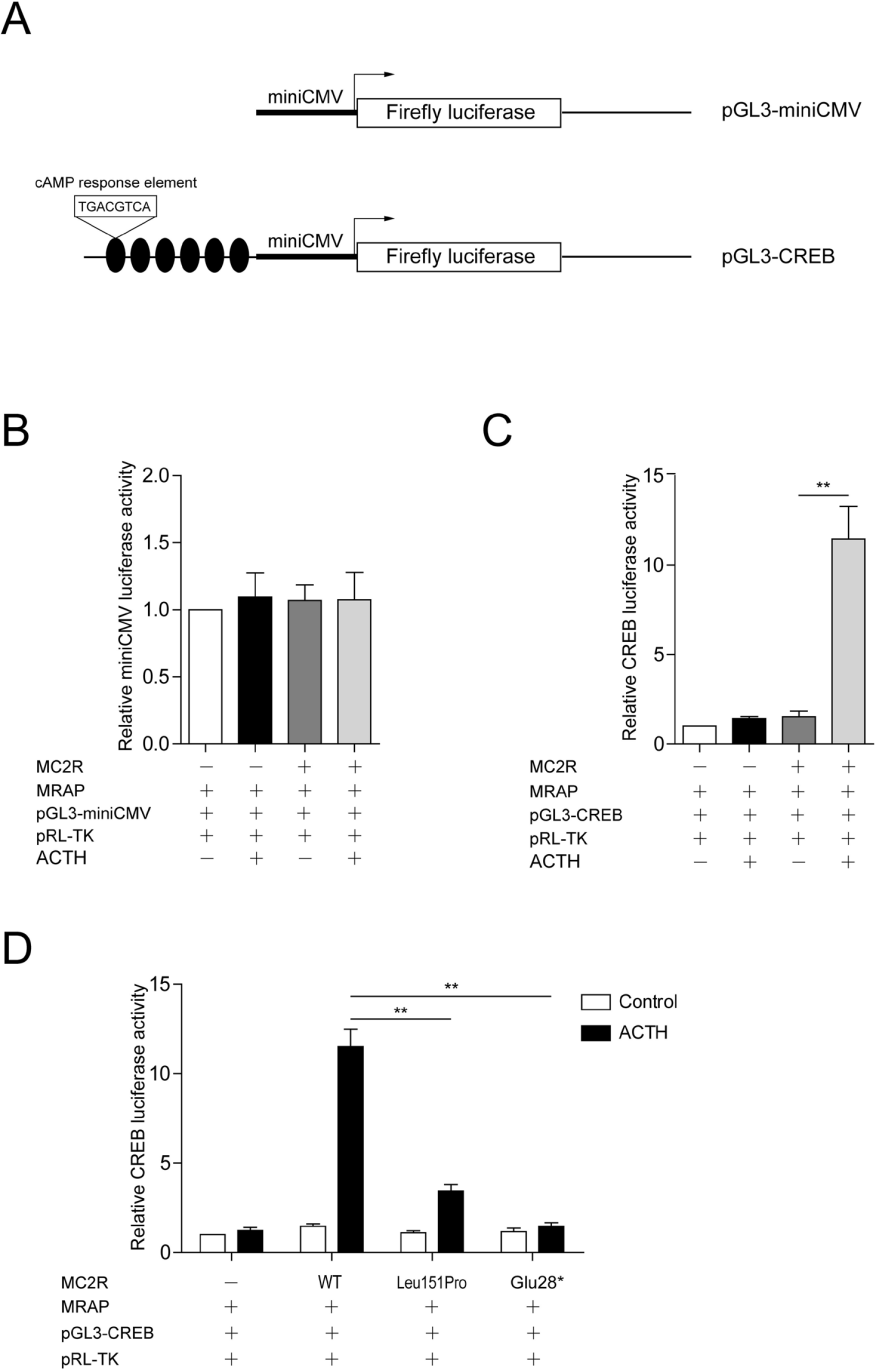
Variants of MC2R attenuated ACTH‐induced cAMP response. (A) Schematic representation of luciferase reporter constructs containing cyclic AMP response elements. Six tandem cAMP response elements were inserted into the pGL3‐miniCMV vector to generate pGL3‐CREB. (B and C) ACTH stimulation induced MC2R‐mediated pGL3‐CREB luciferase activity, while pGL3‐miniCMV served as a negative control. HEK293T cells were co‐transfected with the indicated vectors, treated with ACTH, and subjected to luciferase assays. Data are presented as mean ± SD. *p* < 0.01, highly significant difference. (D) MC2R variants attenuated ACTH‐induced cAMP response. HEK293T cells were co‐transfected with the indicated vectors, treated with ACTH, and analyzed for luciferase activity. Data are presented as mean ± SD. *p* < 0.01, highly significant difference. All experiments were performed in three independent replicates.

Binding of adrenocorticotropic hormone (ACTH) to MC2R activates the heterotrimeric G protein Gs, leading to cyclic adenosine monophosphate (cAMP) production and subsequent steroidogenesis. To investigate the functional impact of wild‐type and mutant MC2R, luciferase reporter constructs containing six tandem cAMP response elements were generated, designated as pGL3‐CREB (Figure 3A). The pGL3‐miniCMV vector was used as a negative control, in which ACTH stimulation did not induce luciferase activity (Figure 3B,C). In contrast, ACTH significantly activated pGL3‐CREB luciferase expression in the presence of wild‐type MC2R (Figure 3D). HEK293T cells were subsequently co‐transfected with pGL3‐CREB and either wild‐type or mutant MC2R, followed by ACTH stimulation and luciferase reporter assay. As shown in Figure 3D, both MC2R variants exhibited a marked reduction in ACTH‐induced cAMP response. These findings indicate that MC2R (p.Leu151Pro) and MC2R (p.Glu28*) represent loss‐of‐function mutations.

## 4 Discussion

Familial glucocorticoid deficiency (FGD) is a rare autosomal recessive disorder classified as a form of primary adrenal insufficiency, characterized by hereditary unresponsiveness to adrenocorticotropic hormone (ACTH). It typically manifests in the neonatal period or early childhood. FGD type 1 (FGD1, OMIM #202200) results from homozygous or compound heterozygous mutations in the melanocortin 2 receptor (*MC2R*, OMIM #607397) gene. This condition is defined by markedly reduced or undetectable serum cortisol levels, accompanied by persistently elevated ACTH concentrations.

The clinical manifestations of FGD1 include hypoglycemia, seizures, skin hyperpigmentation, recurrent infections, fatigue, failure to thrive, hyperbilirubinemia, cholestasis, hepatitis, coma, and tall stature (Akin et al. 2011; Elias et al. 2000; Lipinski et al. 2018). Certain dysmorphic features, such as a prominent forehead with hypertelorism, and a broad nasal bridge with small, tapering fingers (Elias et al. 2000; Slavotinek et al. 1998), have also been reported. Additionally, children with FGD1 may present with isolated endocrine abnormalities, including thyroid dysfunction and delayed pubic hair development.

To date, approximately 110 patients with FGD1 and more than 60 variants in MC2R have been reported (Heshmatzad et al. 2020). The present case represents the third reported FGD1 patient in mainland China. She was diagnosed with FGD1 in the neonatal period and has been treated with hydrocortisone.

During follow‐up, our patient exhibited increased height SDS at 2.5 years of age, along with facial features including a broad nasal bridge and hyperopia. Although tall stature is not a universal feature of FGD, a subset of patients with FGD1 may exhibit abnormally high stature despite having normal growth hormone levels, sometimes accompanied by macrocephaly and advanced bone age (Arslan et al. 2026; Elias et al. 2000). Following glucocorticoid replacement therapy, the growth rate of these patients may normalize (Duan et al. 2023). In our case, the patient's height SDS decreased from +2.86 at 2.5 years to +0.24 at 6 years, suggesting a transient growth acceleration. The underlying mechanisms remain incompletely understood. It has been proposed that chronically elevated ACTH levels may influence growth through interactions with other melanocortin receptors expressed in the bone and cartilage growth plates; however, this hypothesis requires further investigation (Tourkova et al. 2017; Zaidi et al. 2010). In vitro experiments have demonstrated that ACTH can enhance the proliferation and differentiation of chondrocyte precursors, thereby stimulating cartilage formation (Evans et al. 2004; Yeh et al. 2006). In addition, reduced cortisol levels may also contribute to altered bone growth regulation (Gabbitas et al. 1996). To date, the GH‐IGF1 axis has been reported as normal in the overwhelming majority of FGD1 patients, with only isolated rare cases describing transient GH insufficiency (Gujral et al. 2019). Serum IGF‐1 levels at 2.5 and 6 years of age were within the age‐appropriate reference ranges (157 and 192 ng/mL, respectively), making growth hormone excess unlikely. These findings highlight the importance of longitudinal growth monitoring in patients with FGD1.

Generally, there are no obvious abnormalities in renin, aldosterone, and angiotensin II levels in FGD1 patients. Patients with salt‐depleted adrenocortical hypoplasia that occur due to severe loss‐of‐function of MC2R mutations are generally rare (Kardas Yildiz et al. 2024; Lin et al. 2007). Individual cases have reported that FGD1 patients might have delayed pubic hair development, and no other sexual signs were found to be affected. Our patient did not exhibit a salt‐loss type of adrenocortical dysfunction or abnormalities in the reproductive system.

The clinical manifestations associated with MC2R mutations are linked to cAMP signaling, as demonstrated in in vitro experiments. Binding of ACTH to MC2R stimulates adenylyl cyclase, leading to cAMP production, PKA activation, and subsequent phosphorylation of specific nuclear factors. These factors bind to target promoters and recruit coactivator proteins to drive steroidogenic gene transcription (Mohammed et al. 2022; Novoselova et al. 2019; Ruggiero and Lalli 2016). Recent studies have established patient‐derived iPSC models carrying MC2R mutations to investigate their functional consequences (Zhang et al. 2024). In this study, we assessed the effects of MC2R mutations on mRNA and protein expression using RT‐qPCR and immunoblotting analyses. Our results showed that one MC2R variant (p.Glu28*) disrupted both mRNA and protein expression, while the other variant (p.Leu151Pro) did not affect mRNA levels but resulted in a decreased protein level (Figure 2C,D). This suggests an indirect effect of the p.Leu151Pro mutation. Since ACTH binding to MC2R induces cAMP production, we further investigated whether these MC2R variants could modulate transcriptional activity via cAMP. The luciferase reporter assay demonstrated that both MC2R variants significantly attenuated the ACTH‐induced cAMP response.

## 5 Conclusion

In our study, functional assays revealed that the MC2R (p.Leu151Pro) and MC2R (p.Glu28*) mutations are loss‐of‐function mutations, which attenuate the ACTH‐induced cAMP response. These findings suggest that these two MC2R variants may contribute to the development of familial glucocorticoid deficiency (FGD). Furthermore, the underlying signaling pathway appears to be a likely mechanism for explaining the phenotypic characteristics observed in FGD syndrome.

## Author Contributions

Conceptualization, Wenli Lu; Methodology, Ni Zhen and Yonghui Tao; Validation, Ni Zhen, Yonghui Tao; Formal Analysis, Ni Zhen and Yonghui Tao; Resources, Wenli Lu; Data Curation, Wenli Lu and Chuanyin Li; Writing – Original Draft Preparation, Ni Zhen, Yonghui Tao; Writing – Review and Editing, Wenli Lu and Chuanyin Li; Visualization, Chuanyin Li; Supervision, Wenli Lu; Project Administration, Wenli Lu; Funding Acquisition, Wenli Lu and Chuanyin Li. All authors have read and agreed to the published version of the manuscript.

## Funding

This work was supported by the Shanghai Sailing Program (Grant number: 19YF1444700), the Interdisciplinary Program of Shanghai Jiao Tong University (Grant number: ZH2018QNA64), and the Primary Health Care Foundation of China (Grant number: BH005417).

## Ethics Statement

This study was approved by the Ethics Committee of Ruijin Hospital (Approval No. 2020‐148). All methods were performed in accordance with the ethical standards as laid down in the Declaration of Helsinki and its later amendments or comparable ethical standards.

## Consent

Written informed consent was obtained from the patient's parents. No animals were used in this study.

## Conflicts of Interest

The authors declare no conflicts of interest.

## Data Availability

All datasets generated and analyzed for this study are included in the article. Relevant data are available from the corresponding author on reasonable request.
